# Role of TRPM7 Channels in Hyperglycemia-Mediated Injury of Vascular Endothelial Cells

**DOI:** 10.1371/journal.pone.0079540

**Published:** 2013-11-01

**Authors:** Huawei Sun, Tiandong Leng, Zhao Zeng, Xiuren Gao, Koichi Inoue, Zhi-Gang Xiong

**Affiliations:** 1 Department of Cardiology, First Affiliated Hospital, Sun Yat-sen University, Guangzhou, China; 2 Neuroscience Institute, Morehouse School of Medicine, Atlanta, Georgia, United States of America; University of Kansas School of Medicine, United States of America

## Abstract

This study investigated the change of transient receptor potential melastatin 7 (TRPM7) expression by high glucose and its role in hyperglycemia induced injury of vascular endothelial cells. Human umbilical vein endothelial cells (HUVECs) were incubated in the presence or absence of high concentrations of D-glucose (HG) for 72h. RT-PCR, Real-time PCR, Western blotting, Immunofluorescence staining and whole-cell patch-clamp recordings showed that TRPM7 mRNA, TRPM7 protein expression and TRPM7-like currents were increased in HUVECs following exposure to HG. In contrast to D-glucose, exposure of HUVECs to high concentrations of L-glucose had no effect. HG increased reactive oxygen species (ROS) generation, cytotoxicity and decreased endothelial nitric oxide synthase protein expression, which could be attenuated by knockdown of TRPM7 with TRPM7 siRNA. The protective effect of silencing TRPM7 against HG induced endothelial injury was abolished by U0126, an inhibitor of the extracellular signal-regulated kinase signaling pathway. These observations suggest that TRPM7 channels play an important role in hyperglycemia-induced injury of vascular endothelial cells.

## Introduction

 Diabetes mellitus is associated with vascular complications that contribute to the increased morbidity and mortality of the disease [1,2]. Vascular endothelial cells are early and primary targets of hyperglycemic damage in diabetes [3]. It has been reported that diabetic patients have increased generation of reactive oxygen species (ROS) [4], and the production of oxygen metabolites is an important trigger for vascular complications [5-7]. The underlying mechanisms, however, are elusive.

 The TRP channels function as cellular sensors for various internal and external stimuli [8]. Among the 28 unique mammalian TRP channel isoforms that have been identified, at least 19 (all of the TRPC; TRPV1, TRPV2, and TRPVV4; all of the TRPM except TRPMM5; and TRPP1 and TRPP2) are expressed in vascular endothelial cells [9-11]. Among these channels, TRPC1, -C4, -C6, and -M7 have been linked to endothelial barrier dysfunction and perturbed angiogenic processes [12]. In addition, TRPC3, -C4, -M2, and -M7 have been suggested to be responsible for oxidative damage and cell death [10,13]. TRPM7 is one of only two vertebrate ion channels to contain both ion channel and kinase domains [14]. It has an important role in oxidative stress-induced cell injury [15-17]. Suppressing TRPM7 expression, for example, decreased Ca^2+^ uptake and increased neuronal viability [16]. Consistent with the notion that conduction of multiple ions are involved in TRPM7’s ability to mediate cell death, overexpression of TRPM7 in HEK (human embryonic kidney) cells increased Mg^2+^ and Ca^2+^ influx, which led to increased production of ROS [18]. Recently, the expression of TRPM7 was shown to be upregulated by H_2_O_2_ in HUVEC, which suggests that high levels of TRPM7 might represent a marker of oxidative stress in these cells [19].

 Although TRPM7 channel has been known to play an important role in neuronal viability and response to cellular stress, there have been no studies focusing on its role in hyperglycemia induced vascular endothelial cell injury. Thus, the current study was designed to investigate 1) the effect of hyperglycemia on TRPM7 expression in HUVECs; and 2) the role of TRPM7 in hyperglycemia-mediated injury of HUVECs.

## Materials and Methods

### Reagents and antibodies

 The following reagents/antibodies were used: transfection reagent lipofectamine^TM^ RNAiMAX (invitrogen); protease inhibitor cocktail (Sigma); phosphatase inhibitor (Roche), mouse monoclonal antibody against TRPM7 (Abcam, cat:ab85016, lot:GR23197-7), mouse monoclonal antibody against endothelial nitric oxide synthase (eNOS) (BD Biosciences); rabbit polyclonal antibodies against phospho-extracellular signal-regulated kinase (ERK) 1/2, phopho-c-Jun N-terminal kinase (JNK), and phospho-p38 mitogen-activated protein kinase (MAPK), phospho-Bcl-2, cleaved Caspase-3 and MEK1/2 inhibitor U0126 (Cell signaling). 

### Cell Culture

 HUVEC were cultured as described [20]. Cells were purchased from Lonza, and grown in EGM-2 medium containing 2% fetal bovine serum and trophic factors (Lonza). Subconfluent cultures were passaged according to a standard trypsinization protocol. They were used for experiments at passages three and six. To evaluate the effects of high glucose on TRPM7 channel expression, HUVECs were exposed to 5.5 mM D-glucose (control) or 30 mM D-glucose (HG) for 72h. For osmotic control, cells were also cultured with 24.5 mM L-glucose plus 5.5 mM D-glucose in the medium.

### RNA interference

 Knockdown of TRPM7 experiments were performed as described [21]. A special siRNA targeting nucleotides 406-426 of human TRPM7 (NM_017672) was synthesized according to the previous study [21]. A non-targeting siRNA (Cat# 12935-112, Invitrogen) was used as a negative control. Briefly, cells were transfected with 30 nM siRNA or control siRNA using transfection reagent lipofectamine^TM^ RNAiMAX (Invitrogen) according to the manufacturer’s instructions. Non-target control siRNA was used as a parallel control. Cells were used 2 days later for experiments.

### RT-PCR and real-time PCR

 Total RNAs were extracted with RNA purification kit (Qiagen) and transcripted to cDNA using superscript^®^ First-strand synthesis system (invitrogen). RT-PCR reactions were performed using GoTag Flex DNA polymerase (Promega). The amplification cycles consisted of denaturation at 95°C for 3 min, 29 cycles of denaturation at 95°C for 45 s, annealing at 60°C for 30 s, and extension at 72°C for 45s. Real-time PCR were performed using SsoAdvanced^TM^ SYBR^®^ Green supermix (Bio-Rad) in C1000^TM^ Thermal cycler (Bio-rad). Real-time PCR reaction was initiated at 95°C for 10 min, followed by 40 cycles of 95°C for 10 s, 60°C for 10 s, 72°C for 15 s. Melting curve analysis was carried out from 65°C to 95°C with a heating rate of 0.1°C for 1 s. Primer sequences were as follows: RT-PCR for TRPM7, Fw, 5′-AGGAGAATGTCCCAGAAATCC, and Rv, 5′-TCCTCCAGTTAAAATCCAAGC; or for beta-actin: Fw, 5'-CATCCTGCGTCTGGACCTG, and Rv, 5'-ATCTCCTTCTGCATCCTGTC. Real- time PCR for TRPM7: Fw, 5′-CTTATGAAGAGGCAGGTCATGG, and Rv, 5′-CATCTTGTCTGAAGGACTG; or for GAPDH: Fw, 5′-AACTGCTTAGCACCCCTGGC, and Rv, 5′-ATGACCTTGCCCACAGCCTT. 

### Western blotting

 Western blotting was performed as described [22]. Cells cultured on 35 mm dishes or 6-well plates were lysed in lysis buffer (50 mM Tris-HCl, pH 7.4, 150 mM NaCl, 1% Triton X-100, 0.5% Sodium deoxycholate, 0.1% sodium dodecyl sulfate, protease and phosphatase inhibitor cocktail). After centrifugation at 13000g at 4°C for 30 min, the lysates were collected. Protein concentration was assessed using Bradford reagent (Bio-Rad). The aliquots were then mixed with Laemmli sample buffer and boiled at 95°C for 10 min. 30 µg total protein was loaded per lane for western blot. The samples were resolved by 10% SDS-PAGE, followed by electrotransfer to polyvinylidene difluoride membranes. For visualization, blots were probed with antibodies against TRPM7 (1:500), eNOS (1:2000), phospho-ERK (1:1000), phospho-p38MAPK (1:500), phospho-JNK (1:1000), or beta-actin (1:2000), and detected using horseradish peroxidase-conjugated secondary antibodies (1:1000; Cell Signaling) and developed by an ECL kit (Millipore). The intensity of the protein band was quantified with Image J software (NIH). 

### Cell viability assay

 Cell viability was determined by 3-[4,5-dimethylthiazol-2-yl]-2,5-diphenyltetrazolium bromide (MTT) incorporation. Endothelial cells were seeded in 96-well culture plates with 200 μl culture medium per well. Cells were cultured to 90% confluence. Four hours before the culture was terminated, 20 μl assay medium containing 5 μg/ml MTT was added to each well. After 4 h of incubation at 37°C, the medium was aspirated and the cells were lysed by addition of 100 μl DMSO. The optical density of each sample was measured in a microplate reader using reference wavelength of 570 nm.

### Cytotoxicity assay

 Cytotoxity was measured by Lactate dehydrogenase (LDH) assay, which was performed as described [23]. Cells grown on 24-well plates were washed with phosphate-buffered saline. 50 μL medium was taken from each well and placed into 96-well plate for background LDH measurement. Cells were then incubated with Triton X-100 (final concentration 0.5%) for 30 min at 37°C. 50 μL of supernatants were withdrawn from each well for maximal LDH measurement. 50 μL of assay reagent from cytotoxicity detection kit (Roche Diagnostics) was added to each sample and mixed. 30 min later, the absorbance at 492 and 620 nm was examined by spectrometer (SpectraMax Plus, Molecular devices), and the values of the absorbance at 492 nm were subtracted by those at 620 nm to yield the value of LDH release.

### Intracellular ROS production assay

 ROS production assay was performed as described [23]. HUVECs were incubated with dye 2’,7’-dichlorofluorescein diacetate (DCF-DA, 50 uM) for 60 min and then washed and resuspended in Hanks’ balanced salt solution. DCF-DA is a non-polar compound that readily diffuses into cells, where it is hydrolyzed to the non-fluorescent polar derivative DCFH and thereby trapped within the cells. In the presence of reactive oxygen species, DCFH is oxidized to the highly fluorescent DCF, which was monitored spectrophotometrically at 530 nm with an excitation wavelength of 485 nm.

### NO metabolite measurement

 The production of nitric oxide (NO) was examined by measurement of nitrite, a stable product of NO, using fluorometric reagent, 2,3-diaminonaphthalene. As described previously [21], 100 µl of samples were transferred to 96-well plate, and incubated in dark with 10 µl fresh 2,3-diaminonaphthalene solution (50 µg/ml in 0.62 N HCl) for 10 min at room temperature. The reactions were terminated with 5 µl of 2.8 N NaOH. The formation of 2,3-diaminonaphthotriazole was measured using fluorescent multi-well plate reader (SpectraMax Gemini, Molecular Devices) with excitation/emission at 365/450 nm. The fluorescence signal was analyzed using SoftMax Pro software.

### Electrophysiology

 Whole-cell voltage-clamp recordings were performed as described [24]. Patch electrodes were constructed from thin-walled borosilicate glass (WPI) and had resistances of 2 to 3 MΩ. Currents were recorded using Axopatch 200B amplifier with pCLAMP software (Axon Instruments). They were filtered at 2 kHz and digitized at 5 kHz using Digidata 1322A. Data were eliminated from statistical analysis when access resistance was >10 MΩ or leak current was >100 pA at -60 mV. A multibarrel perfusion system was used to achieve a rapid exchange of external solutions. Standard extracellular solution contained (in millimolar) 140 NaCl, 5.4 KCl, 2 CaCl_2_, 1 MgCl_2_, 20 HEPES, 10 glucose (pH 7.4 adjusted with NaOH; 320-335 mOsm). For the induction of TRPM7 current, Ca^2+^ and Mg^2+^ free standard extracellular solution was applied. Patch electrodes contained (in millimolar): 140 CsF, 10 HEPES, 1 CaCl_2_, 11 EGTA, 2 TEA, (pH 7.25 adjusted with CsOH, 290-300 mOsm). All experiments were done at room temperature.

### Statistics

**Figure 1:**
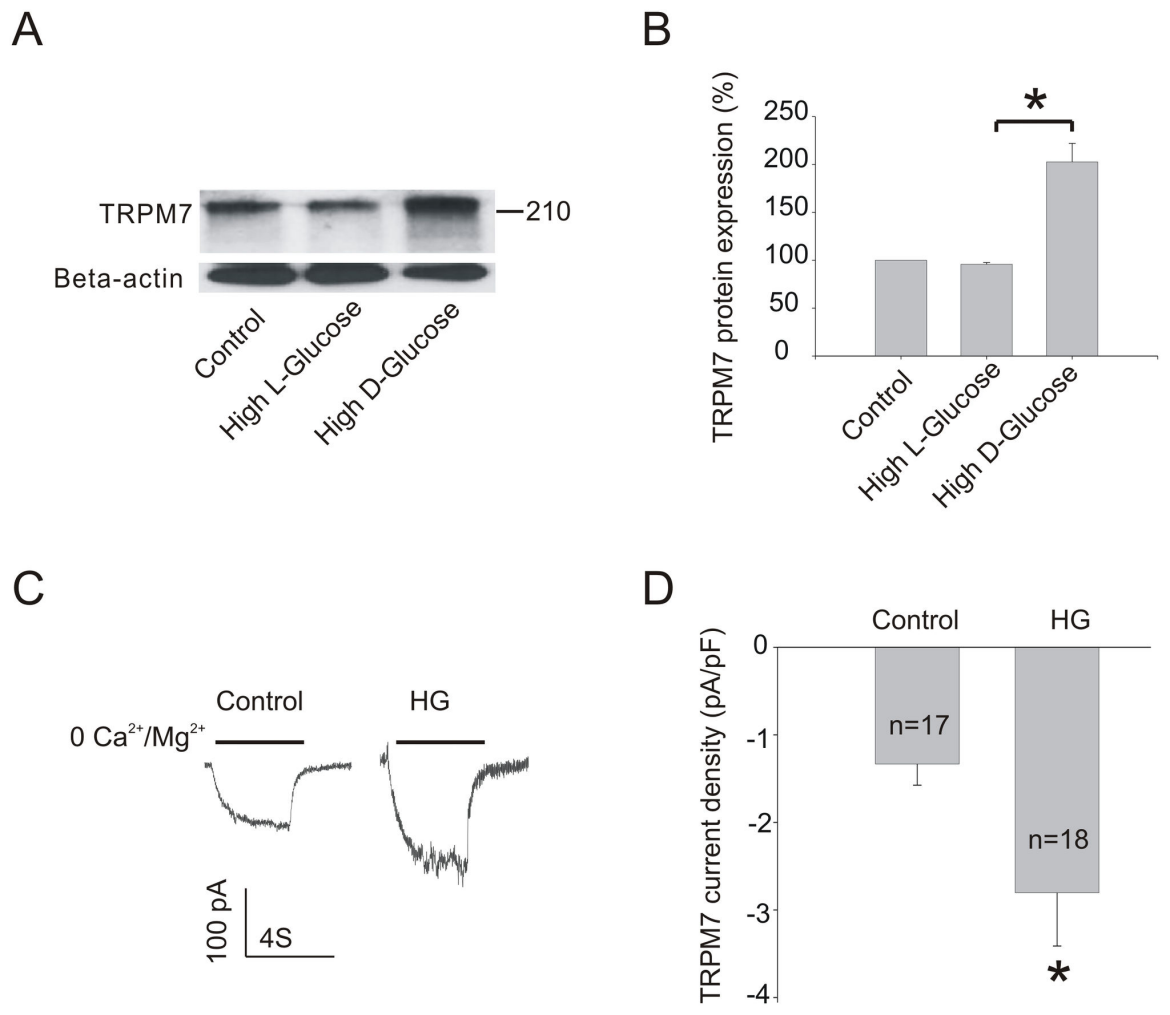
Effect of HG on TRPM7 protein expression in HUVECs. (A) Representative immunoblots showing TRPM7 protein expression in HUVECs with or without HG (30 mM) for 72h. (B) The corresponding bar graphs showing relative expression of TRPM7 protein normalized to beta-actin. (C) Representative TRPM7-like currents recorded in HUVECs cultured in control or HG (30 mM) for 72h. (D) TRPM7-like current density. **p<0.01 vs. HG; *p<0.05 vs. control. n=4 for immunoblotting and 17-18 cells patched for current recording.

Data are expressed as the mean ± SEM, and analyzed using one-way ANOVA with or without post-hoc multiple comparison tests. Data in Figure 1D and Figure S3 were analyzed by Student’s *t*-test. A *p* value of <0.05 was considered significant.

## Results

### Effect of high D-glucose on TRPM7 protein expression in HUVECs

 Considering the existing problems of some TRPM7 antibodies, we checked the TRPM7 antibodies using HEK-293 cells overexpressing TRPM7 prior to this study. We found that mouse monoclonal anti-TRPM7 antibody (Abcam, cat:ab85016, lot:GR23197-7) has a better quality (Figure S1) than goat polyclonal anti-TRPM7 antibody (Abcam, ab729) (data not shown). Therefore, the mouse monoclonal antibody against TRPM7 (Abcam, cat:ab85016, lot:GR23197-7) was used for this study. We then examined the effect of HG on TRPM7 protein expression in HUVECs. HUVECs were exposed to 30 mM D-glucose or 24.5 mM L-glucose (plus 5.5 mM D-glucose in basic medium) for 72h. As shown in Figure 1A and B, the administration of HG significantly increased TRPM7 protein expression by two fold compared with control conditions (control: 100, HG: 202.6±19.5, *p*<0.05; n=4). The effect of HG on TRPM7 protein expression in HUVEC was not attributable to hyperosmolality of the medium, since addition of high concentration of L-glucose did not produce any change in the expression of TRPM7 protein (control: 100, high L-glucose: 95.8±1.8, *p*>0.05; n=4). Consistent with the Western blot data, whole-cell patch-clamp recordings demonstrated that exposure to HG increased amplitude of TRPM7-like current in HUVECs (control: n=17, HG: n=18, *p*<0.05) (Figure 1C and D).

### Effect of TRPM7 siRNA on TRPM7 mRNA and Protein Expression in HG Treated HUVECs

**Figure 2:**
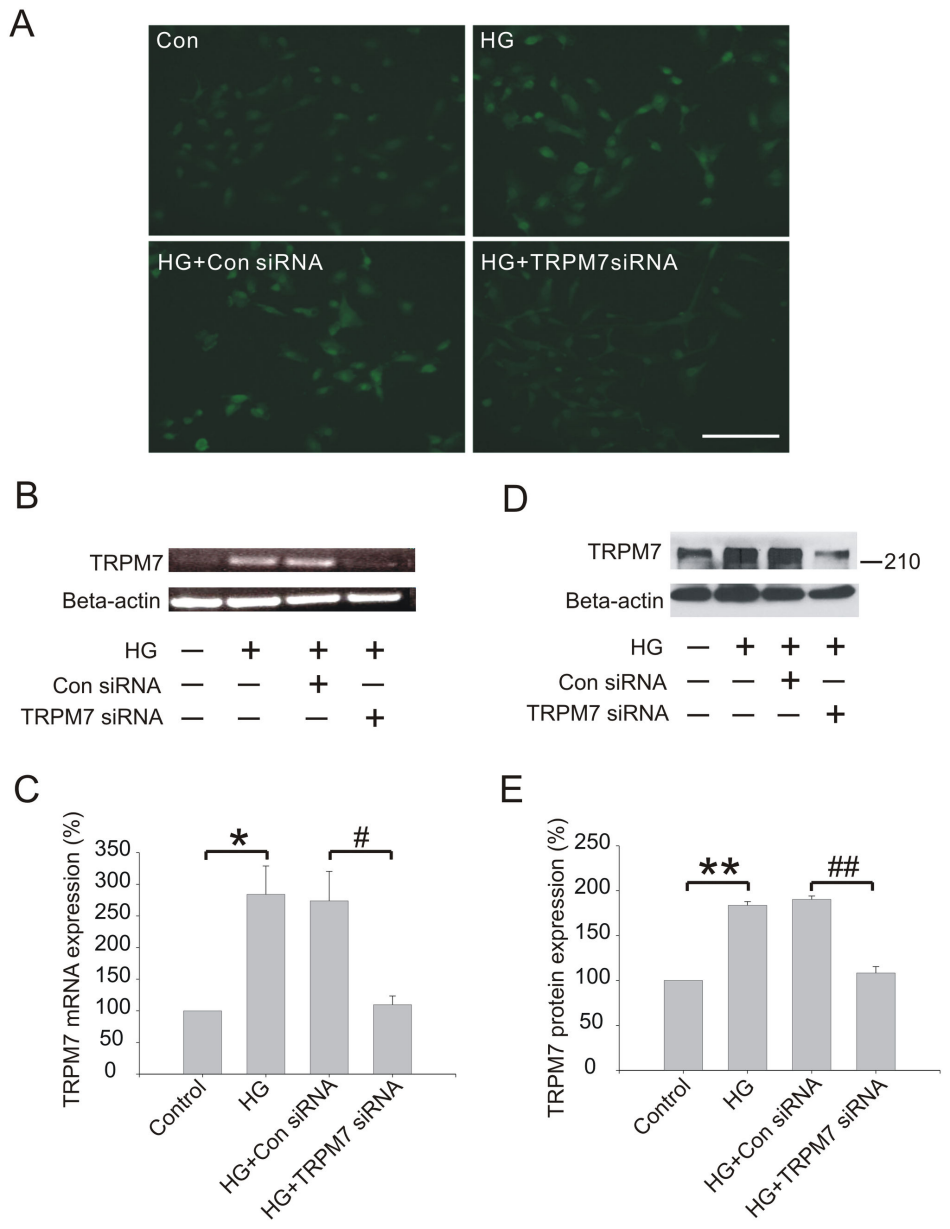
Effect of TRPM7 siRNA on TRPM7 mRNA and protein expression in high D-glucose (HG, 30 mM) treated HUVECs. The cells were preincubated with TRPM7 siRNA or control siRNA for 48h, and then stimulated with HG for 72h. (A) TRPM7 protein level measured by immunofluorescence. *Scale bar, 100 µm*. (B) TRPM7 mRNA level detected by RT-PCR. (C) TRPM7 mRNA level normalized to GAPDH. (D) Representative immunoblots showing the level of TRPM7 protein expression. (E) The corresponding bar graphs showing the relative expression of TRPM7 protein normalized to beta-actin. **p<0.01 vs. control; ^#^ *p*<0.05 vs. control siRNA, ^##^ *P*<0.01 vs. control siRNA. n=4 for RT-PCR and immunoblotting.

 To assess the effect of TRPM7 siRNA on TRPM7 mRNA and protein expression in HG treated HUVECs, cells were preincubated with TRPM7 siRNA or control siRNA for 48 h, and then exposed to HG (30 mM) for 72 h. Consistent with the findings mentioned above, immunofluorescence staining (Figure 2A) and Western blotting (Figure 2D and E) showed that TRPM7 protein expression was significantly up-regulated in HG treated HUVECs compared with control cells (control: 100, HG: 183.7±4.3, *p*<0.01; n=4). TRPM7 siRNA reduced TRPM7 protein expression in HG treated HUVEC by more than 40% compared with control siRNA (control siRNA: 190.3±3.8, TRPM7 siRNA: 108.3±7.2, *p*<0.01; n=4). RT-PCR (Figure 2B) and Real-time RCR (Figure 2C) results showed that TRPM7 mRNA expression was up-regulated to 2.84 fold in HG treated HUVECs (control: 100, HG: 284±44.8, *p*<0.05; n=4), and that the TRPM7 mRNA expression in HG treated HUVECs was down-regulated by TRPM7 siRNA (control siRNA: 273.7±46.5, TRPM7 siRNA: 109.7±13.8, *p*<0.05; n=4). 

### Effect of silencing TRPM7 on cell viability, cytotoxicity in HG treated HUVECs

**Figure 3:**
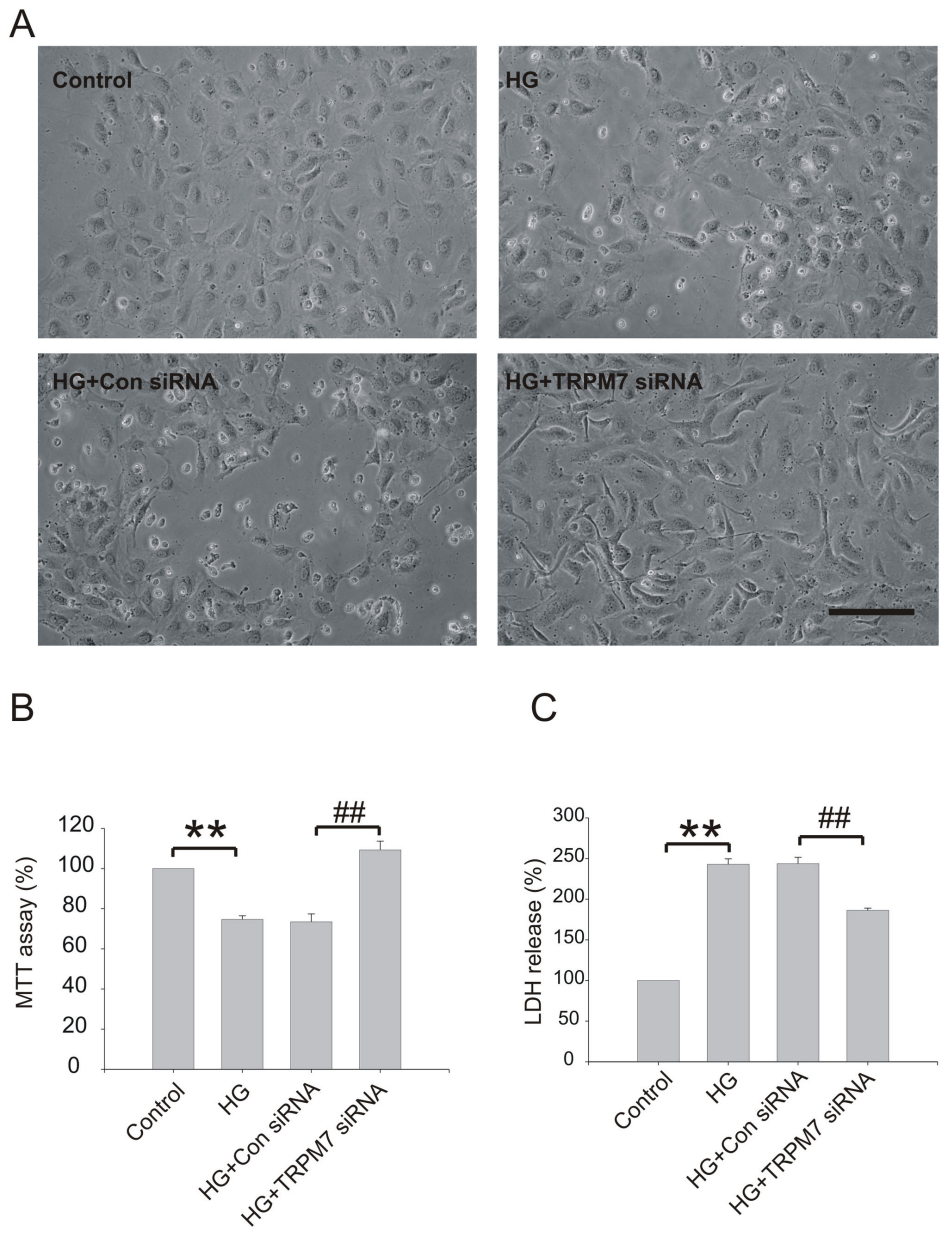
Effect of TRPM7 siRNA on viability and cytotoxicity in HG treated HUVECs. The cells were preincubated with TRPM7 siRNA or control siRNA for 48h, and then stimulated with HG for 72h. (A) Confluent field by light microscopy. *Scale bar, 100 µM*. (B) Cell viability was assessed by MTT assay. (C) Cytotoxicity was assessed by LDH release assay. **p<0.01 vs. control; ^##^ *p*<0.01 vs. control siRNA. n=5 for MTT and LDH assay.

 To further examine the effect of TRPM7 siRNA on cell viability in HG treated HUVECs, cells were preincubated with TRPM7 siRNA or control siRNA for 48h, and then stimulated with HG for 72h. As shown in Figure 3A-C, HG exposure dramatically increased cytotoxicity as shown by morphological change, such as cell body shrinkage (Figure 3A), decreased cell viability as shown by MTT assay (control: 100, HG: 74.7±1.8, *p*<0.01; n=5) (Figure 3B) and increased cell injury by LDH assay (control: 100, HG: 242.9±6.7, *p*<0.01; n=5) (Figure 3C). Silencing TRPM7 alleviated HG induced cytotoxicity (control siRNA: 243.7±7.8, TRPM7 siRNA: 186.1±2.8, *p*<0.01, n=5), and increased cell viability (control siRNA: 73.4±3.9, TRPM7 siRNA: 109.2±4.4, *p*<0.01; n=5) (Figure 3B and C). Our previous study showed that silencing TRPM7 caused a slight proliferation in normal concentration of glucose [21], which may contribute to the slight increase in MTT absorbance following TRPM7 siRNA treatment. In the current study, we further compared the MTT absorbance in the normal and high glucose conditions with or without TRPM7 knockdown (Figure S2). We found that TRPM7-siRNA caused a ~6% increase of the MTT absorbance in normal glucose conditions, which is consistent with our previous observation that TRPM7 knockdown causes a slight increase of proliferation in HUVECs. However, in high glucose conditions, TRPM7-siRNA caused more than 30% increase of the MTT absorbance in comparison with the Con-siRNA (Figure S2, p<0.01, n=6). In combination with the morphological observation and LDH release assay, this data suggests that TRPM7-siRNA induced increase of the MTT absorbance in high glucose conditions is largely dependent on the increase of the survivability of HUVECs. 

 High glucose treatment induces apoptosis in HUVEC (26,36). The present observation of the shrinkage of the HUVECs in high glucose treatment is consistent with apoptosis. Thus, we checked the level of the pro- and anti-apoptosis player Caspase-3 and Bcl-2. We found a higher cleaved Caspase-3 level and a lower phospho-Bcl-2 level in “HG+control siRNA” compared with “HG+TRPM7 siRNA” (Figure S3), which suggests that anti-apoptosis might be one of the mechanisms underlying the protective activity of TRPM7 knockdown in HUVECs. 

### Effect of silencing TRPM7 on eNOS protein expression, NO and ROS generation in HG treated HUVECs

**Figure 4:**
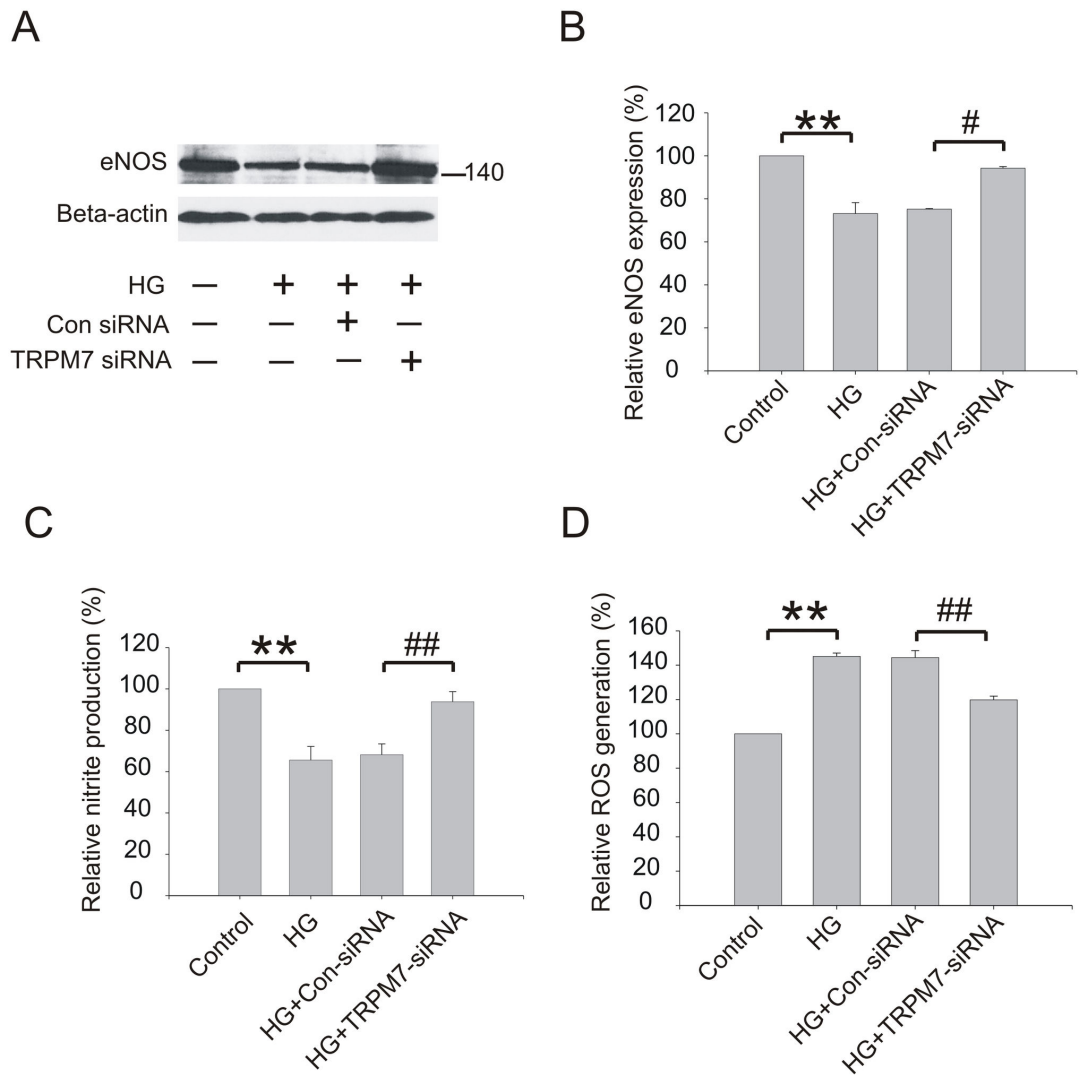
Effect of TRPM7 siRNA on eNOS protein expression, NO and ROS generation in HG treated HUVECs. The cells were preincubated with TRPM7 siRNA or control siRNA for 48h, and then stimulated with HG for 72h. (A) Representative immunoblots showing eNOS protein expression level. (B) The corresponding bar graphs showing the relative expression of eNOS protein normalized to beta-actin. (C) The production of NO was determined by measurement of nitrite, a stable product of NO. (D) The production of intracellular ROS was assessed by the oxidation of 2’,7’-dichlorofluorescin diacetate to fluorescent 2’,7’-dichlorofluorescein. **p<0.01 vs. control; ^##^ *p*<0.01 vs. control siRNA. n=3 for immunoblotting , 5 for ROS generation assay and 6 for NO measurement.

 As shown in Figure 4A and B, eNOS protein expression was down-regulated at 72 h following the exposure to HG (control: 100, HG: 73.2±5.1, *p*<0.01; n=3). Silencing TRPM7 ameliorated the reduction of eNOS protein expression induced by HG (control siRNA: 75.2±0.3, TRPM7 siRNA: 94.2±0.8, *p*<0.05, n=3). To investigate whether TRPM7 knockdown could prevent the reduction of NO production, we measured nitrite, a stable product of NO. As shown in Figure 4C, the production of nitrite was decreased by HG treatment (control: 100, HG: 65.58±6.56, *p*<0.01; n=6), and that silencing TRPM7 ameliorated the reduction of nitrite production (Figure 4C, control siRNA: 68.16±5.22, TRPM7 siRNA: 93.77±4.91, *p*<0.01, n=6).

 Next we determined whether TRPM7 is involved in increased ROS generation in hyperglycemic condition. As reported previously [25], HG increased ROS generation (control: 100, HG: 145.1±1.9, *p*<0.01; n=5). Silencing TRPM7 significantly decreased ROS generation (control siRNA: 144.4±4.1, TRPM7 siRNA: 119.8±2.2; *p*<0.01, n=5, Figure 4D), indicating that activation of TRPM7 channels is involved in hyperglycemia-mediated increase of ROS.

### Effect of silencing TRPM7 on MAPK pathway in HG treated HUVECs

**Figure 5:**
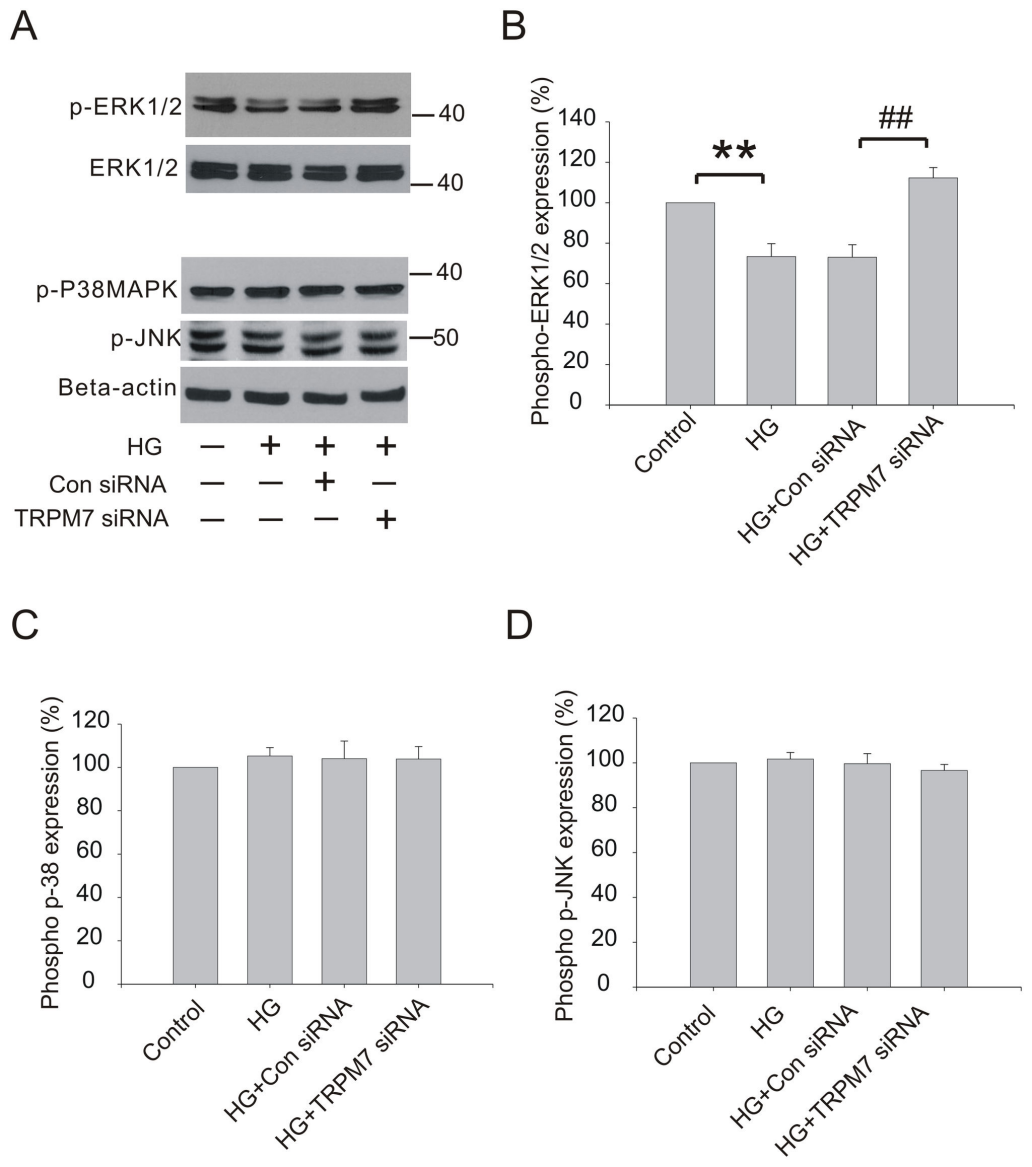
Effect of TRPM7 siRNA on MAPK pathway in HG treated HUVECs. The cells were preincubated with TRPM7 siRNA or control siRNA for 48h, and then stimulated with HG for 72h. (A) Representative immunoblots showing phospho-ERK1/2, phospho-p38MAPK and phospho-JNK protein expression level. (B) The corresponding bar showing the relative optical density of phospho-ERK1/2 protein normalized to total ERK1/2. **p<0.01 vs. control; ^##^ *p*<0.01 vs. control siRNA. n=5 for immunoblotting (C) The corresponding bar graphs showing the relative optical density of phospho-p38MAPK protein normalized to beta-actin. (D) The corresponding bar graphs showing the relative expression of phospho-JNK protein normalized to beta-actin. n=4.

 HG is known to affect MAPK pathway [26]. To determine whether TRPM7 channels are involved in HG-mediated changes in MAPK pathway, we studied the effect of TRPM7 knockdown on the expression of MAPK family including phospho-ERK, phospho-JNK and phospho-p38 MAPK in HG treated HUVECs. Cells were transfected with TRPM7 siRNA or control siRNA for 48h, then stimulated with HG for 72h. As shown in Figure 5A and B, HG decreased phospho-ERK1/2 protein expression in HUVECs at 72h (control: 100, HG: 73.39±6.35, *p*<0.01, n=5). Pretreatment of HUVECs with TRPM7 siRNA prevented the reduction of phospho-ERK1/2 protein expression (control siRNA: 73±6.22, TRPM7 siRNA: 112.26±5.12, *p*<0.01, n=5). In contrast to phospho-ERK1/2, there was no significant change in the expression of phospho-JNK and phospho-p38 MAPK compared with control (Figure 5C and D, n=4). 

### Effect of U0126 on the change of cell viability by TRPM7 siRNA

**Figure 6:**
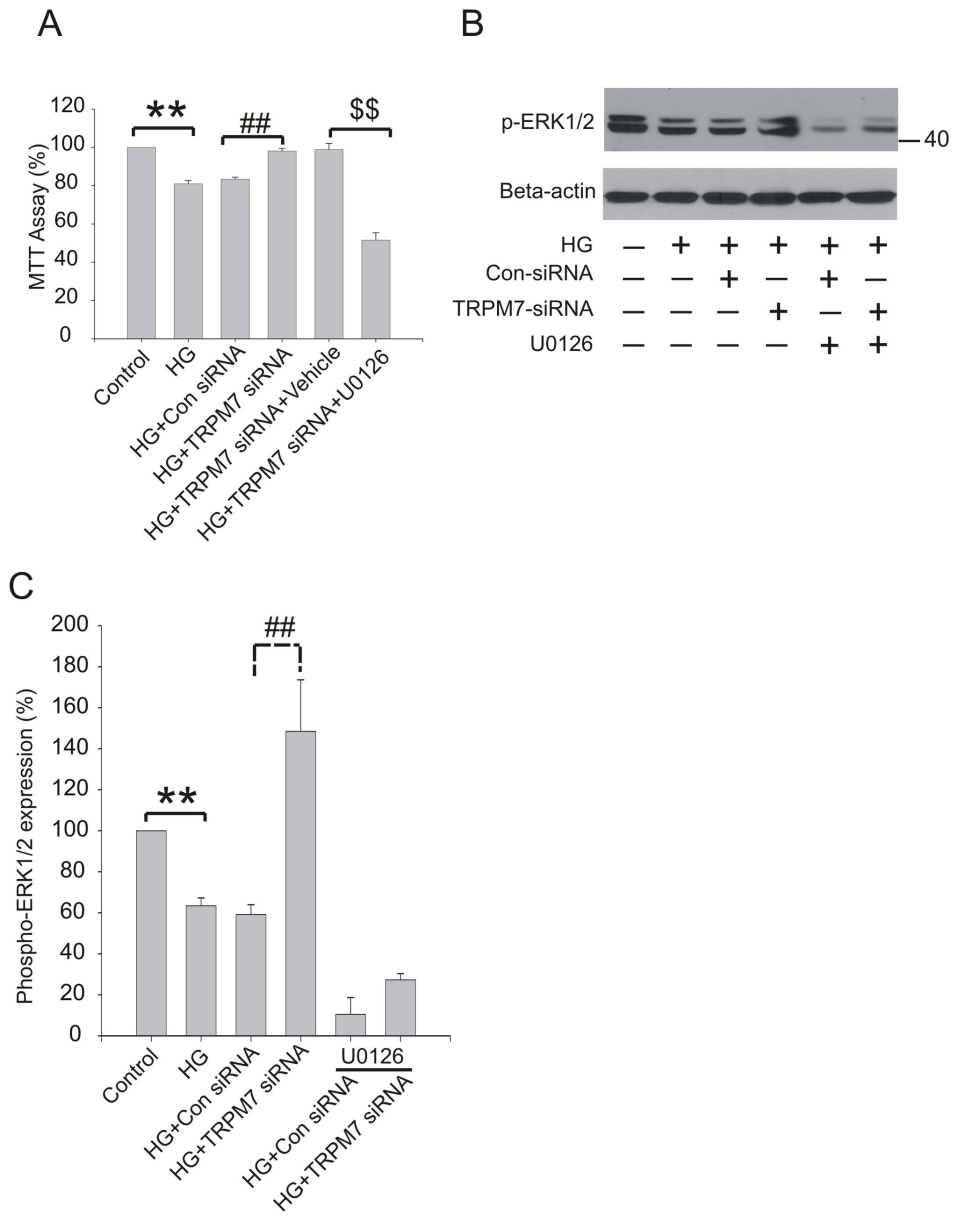
Effect of U0126 on viability and phospho-ERK1/2 expression in HG treated HUVECs. The cells were preincubated with U0126 (10 μM) for 18h, then treated with TRPM7 siRNA for 48h, and finally stimulated with HG for 72h. (A) Cell viability was assessed by MTT assay. (B) Representative immunoblots showing phospho-ERK1/2 expression level. (C) The corresponding bar graphs showing the relative expression of phospho-ERK1/2 protein normalized to beta-actin. **p<0.01 vs. control; ^##^ *p*<0.01 vs. control siRNA. n=5 for immunoblotting.

 MEK1/2 is an ERK kinase, which phosphorylates ERK resulting in its activation. To further elucidate the role of ERK pathway in the protective effect against HG by silencing TRPM7, U0126, an inhibitor of MEK1/2, was used. U0126 (at a concentration of 10 µM) was added to the culture medium. After 18h, cells were treated with TRPM7 siRNA or control siRNA and then stimulated with HG for 72h. As shown in Figure 6A and B, HG increased cell cytotoxicity, while silencing TRPM7 decreased cell cytotoxicity, which was consistent with the above mentioned result (e.g. Figure 3). Following the treatment of U0126, the protective effect by silencing TRPM7 in HG treated HUVECs was almost completely eliminated as demonstrated by MTT assay (Figure 6A). Consistent with the result above (Figure 5), HG decreased phospho-ERK1/2 protein expression while silencing TRPM7 increased phospho-ERK1/2 protein expression (Figure 6B and C). Pretreatment with U0126 dramatically decreased phospho-ERK1/2 protein expression in HUVECs (Figure 6B and C). 

## Discussion

 Our present data show that high D-glucose causes increased TRPM7 protein expression, while high L-glucose had no effect on TRPM7 expression under the same condition. This result implies that the increased TRPM7 protein expression is specific to high D-glucose, and that it is not due to the change of osmolality. This finding is consistent with a previous in vitro study showing high D-glucose induced increase of TRPM7 expression in human monocytes [17], and an in vivo study showing increased TRPM7 mRNA expression in endothelial cells of type I diabetic mice [27]. 

Similar to other TRPs, TRPM7 can be activated by numerous stimuli, including pressure, shear stress, oxidative stress, and intracellular cations, as well as ligand-receptor interactions, indicating the physiological importance of TRPM7 in cellular functions [28-30]. Considering its activation by oxidative stress, the stimuli able to produce an increase in ROS should activate TRPM7, which likely participate in the cell death process. In addition to the direct activation of TRPM7, whether ROS could increase TRPM7 expression is largely unknown. Interestingly, a recent study shows that H_2_O_2_ could increase TRPM7 expression in HUVECs (19), likely through an increased production of ROS. In combination of this finding and ours, we speculate that increased ROS production and oxidative stress in hyperglycemia conditions might be responsible for the increased expression of TRPM7 in HUVECs. 

 The other important finding in the current study is that increased expression of TRPM7 is associated with high D-glucose induced endothelial cell injury. This is supported by the finding that silencing TRPM7 produces a protective effect against high glucose induced cell injury. It is well-known that high D-glucose can cause down-regulation of eNOS expression, increased ROS generation, and cell cytotoxicity [31-35]. The detailed mechanism underlying HG induced cell injury, however, is still unclear. Some studies showed that eNOS deficiency may contribute to diabetic vascular complications in both experimental models and humans, while eNOS enhancer reduces oxidative stress and restores endothelial function in diabetic mice [3,5,32]. Ho and colleagues, for example, found that the expression of eNOS after HG treatment was briefly increased at 6h but gradually decreased after 24h and 48h [3,6]. The regulation of eNOS is complex. In addition to be regulated through protein-protein interaction by some molecules including Caveolin-1 and HSP90, eNOS can be regulated by multiple phosphorylation sites at tyrosine, serine and threonine residues. The regulation of eNOS through phosphorylation by different kinases at different sites is far more complex, which could result in opposing effects on eNOS activity, for example, Y83 phosphorylation by Src-kinase activates the enzyme (37) while Y657 phosphorylation by proline-rich tyrosine kinase 2 appears to attenuate eNOS activity (38). Other studies have shown that eNOS protein level is corresponding to its activity for NO synthesis (39-42). Consistently, our previous study showed that TRPM7 knockdown increase eNOS level and NO production (21). In the current study, we found that TRPM7 knockdown could prevent high glucose-induced decrease of eNOS and NO. In combination with the above studies, we speculate that preserving the eNOS level by TRPM7 knockdown might be responsible for the prevention of NO decrease in HUVECs under high glucose conditions. Thus, increasing eNOS expression and lowering the level of ROS by silencing TRPM7 should be beneficial in protecting against HG induced HUVECs injury. Consistent with the findings in HUVECs, suppressing TRPM7 expression lowered ROS level in other types of cells [13,16]. Thus, lowering ROS level could be a common mechanism for cell protection. Because TRPM7 is permeable to multiple cations such as Ca^2+^, Mg^2+^, and Zn^2+^, further studies might be required to define the involvement of specific ions in the process. Moreover, since TRPM7 channel also contains a kinase domain [14], it is not clear whether the protective effect by silencing TRPM7 is related to the decrease in kinase activation.

 Based on the knowledge that MAPK family plays an important role in physiological and pathophysiological processes and that silencing TRPM7 affects the MAPK pathways [1,2,13], we examined whether and which MAPK pathway is involved in the protective effect against high D-glucose induced HUVECs injury by silencing TRPM7. Our studies suggest that ERK, but not p38 MAPK or JNK pathway, is involved. Activation of ERK1/2 is known to induce changes in gene expression that promotes growth, differentiation and mitosis. Exposure to high levels of D-glucose has been shown to decrease the expression of ERK 1/2 in bovine aortic endothelial cells [43]. We showed a similar effect of high glucose on ERK expression in HUVECs. Interestingly, high glucose-induced reduction of ERK 1/2 expression could be prevented by TRPM7 silencing. Consistent with our previous study of HUVECs in the normal condition [1,2], we found that silencing TRPM7 increased ERK phosphorylation in HUVECs under hyperglycemic condition. In addition we found that the protective effect by silencing TRPM7 against HG induced cell injury could be attenuated by U0126, further supporting the involvement of ERK pathway in HG induced, TRPM7-mediated, injury of HUVECs. The exact mechanism of how TRPM7 silencing increases the expression and activation of ERK signaling is not clear. It might be conceivable that compensatory changes in response to reduced TRPM7 channel activity and the decreased level of intracellular Ca^2+^/Mg^2+^ might result in an increased ERK activation [1,2]. Future studies may determine whether a change in the kinase activity of TRPM7 influences the ERK signaling pathway. 

 In conclusion, we provided the evidence that high D-glucose increases TRPM7 expression in HUVECs which plays an important role in cell injury. Targeting TRPM7 channels might be a novel therapeutic strategy for vascular complications in diabetes.

## Supporting Information

Figure S1 **The expression of TRPM7 in HEK-293 cells in the presence or absence of tetracycline.** HEK-293 cells with inducible expression of TRPM7 were used. These cells were treated with 1 µg/ml tetracycline for 2 days for the induction of TRPM7 expression. Following that, TRPM7 protein levels were examined by immunoblotting using mouse monoclonal antibody against TRPM7 (Abcam, cat:ab85016, lot:GR23197-7).
(TIF)

Figure S2 **Effect of TRPM7 siRNA on the viability of HUVECs in the presence or absence of high glucose (HG) treatment.** The cells were preincubated with TRPM7 siRNA or control siRNA for 48h, and then stimulated with or without HG for 72h. Cell viability was assessed by MTT assay. **p<0.01 n=6.
(TIF)

Figure S3 **Effect of TRPM7 siRNA on the expression of cleaved Caspase-3 and phospho-Bcl-2 in HG treated HUVECs.** The cells were preincubated with TRPM7 siRNA or control siRNA for 48h, and then stimulated with HG for 72h. Following that, Western blotting analysis was performed. (A) Representative blots and (B) Summary data showing the expression of cleaved Caspase-3 and phospho- Bcl-2. Data are mean ± S.E. (n=3, **p<0.01).
(TIF)
