# Supplementary figures and images for: Role of TRPM7 Channels in Hyperglycemia-Mediated Injury of Vascular Endothelial Cells
Source: PLoS One. 2013 Nov 1;8(11):e79540. doi: 10.1371/journal.pone.0079540 (PMC3815131; doi:10.1371/journal.pone.0079540)

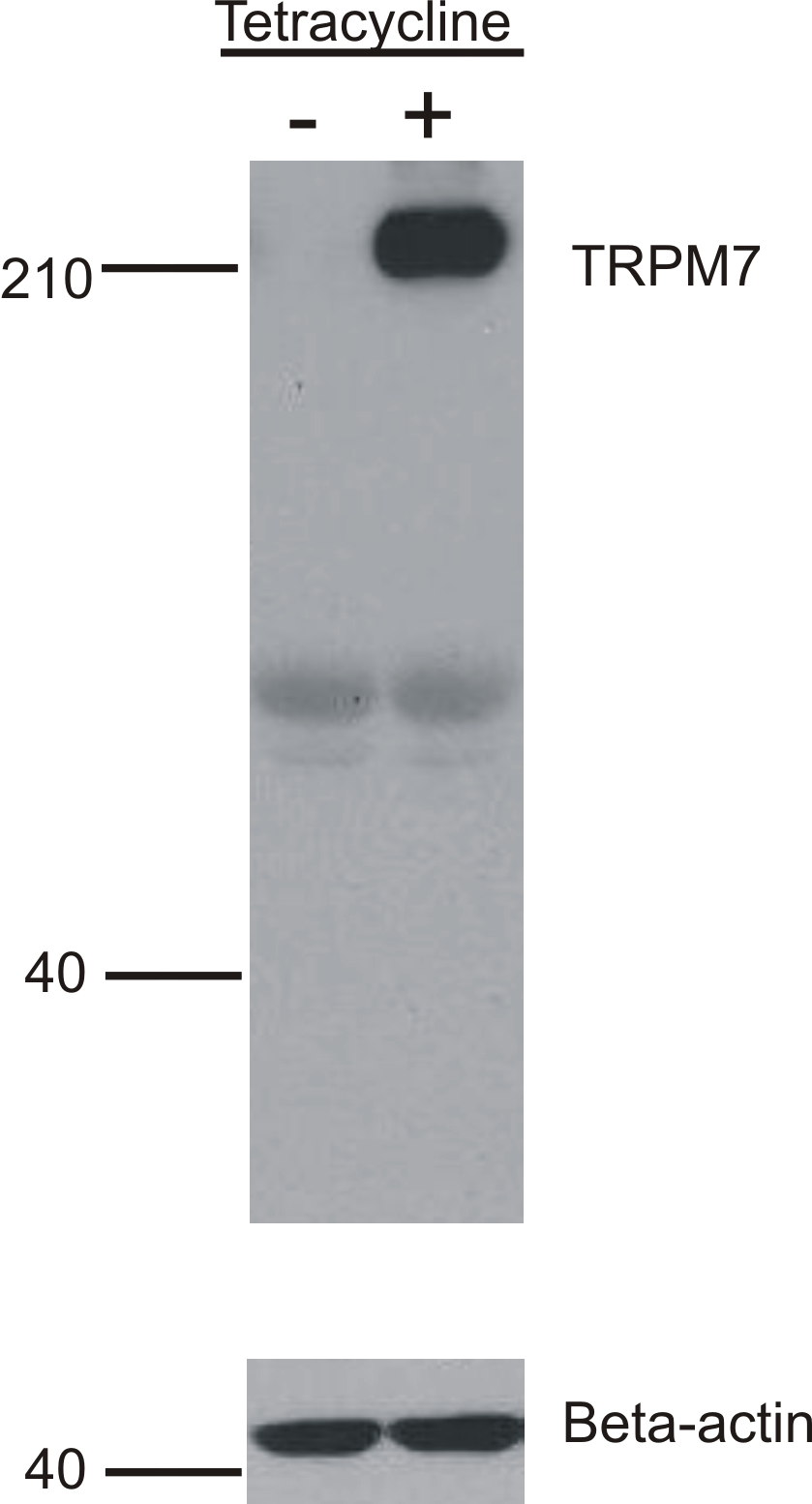

Supplement: Figure S1 — The expression of TRPM7 in HEK-293 cells in the presence or absence of tetracycline. HEK-293 cells with inducible expression of TRPM7 were used. These cells were treated with 1 µg/ml tetracycline for 2 days for the induction of TRPM7 expression. Following that, TRPM7 protein levels were examined by immunoblotting using mouse monoclonal antibody against TRPM7 (Abcam, cat:ab85016, lot:GR23197-7). (TIF) [file pone.0079540.s001.tif]

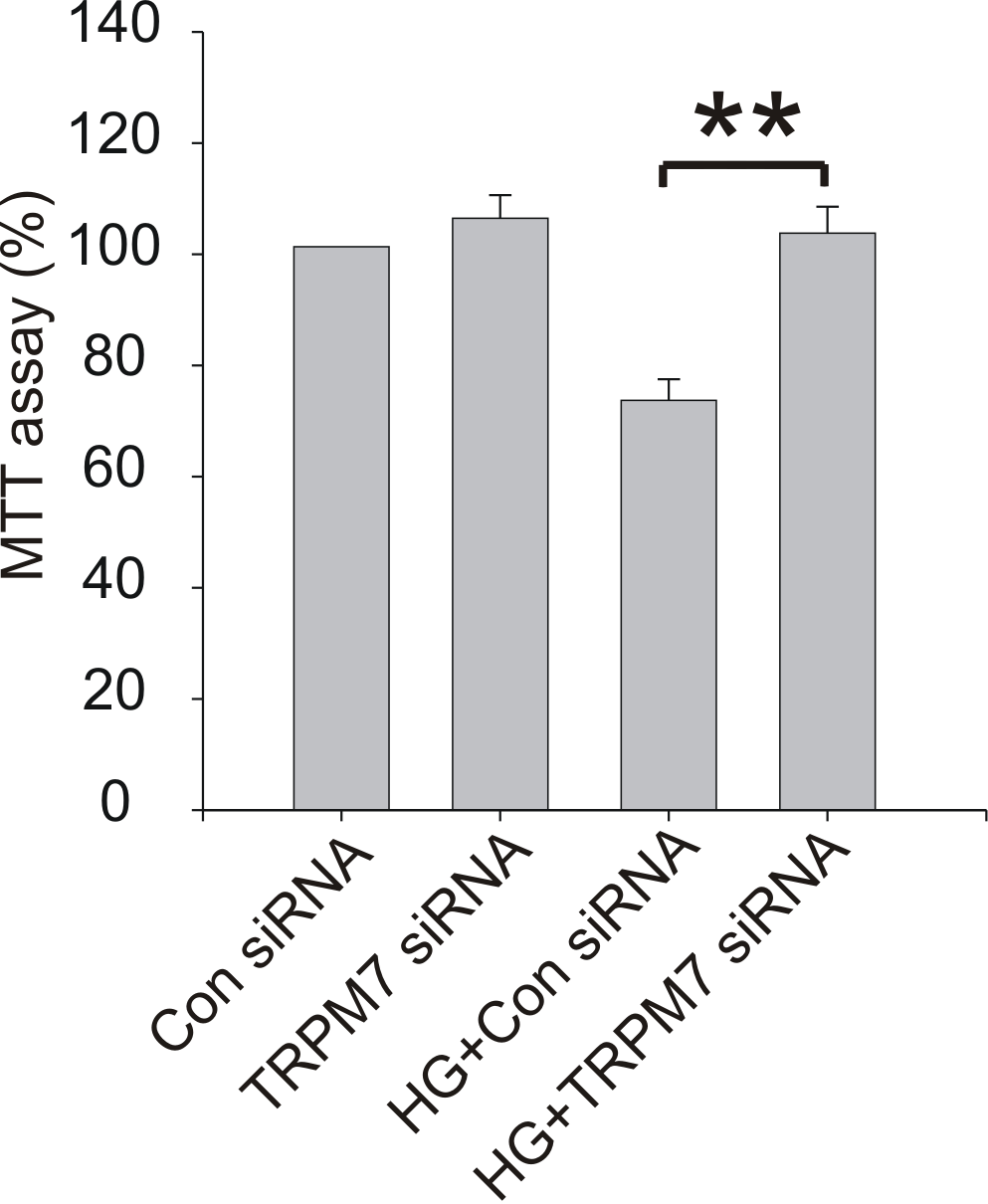

Supplement: Figure S2 — Effect of TRPM7 siRNA on the viability of HUVECs in the presence or absence of high glucose (HG) treatment. The cells were preincubated with TRPM7 siRNA or control siRNA for 48h, and then stimulated with or without HG for 72h. Cell viability was assessed by MTT assay. **p<0.01 n=6. (TIF) [file pone.0079540.s002.tif]

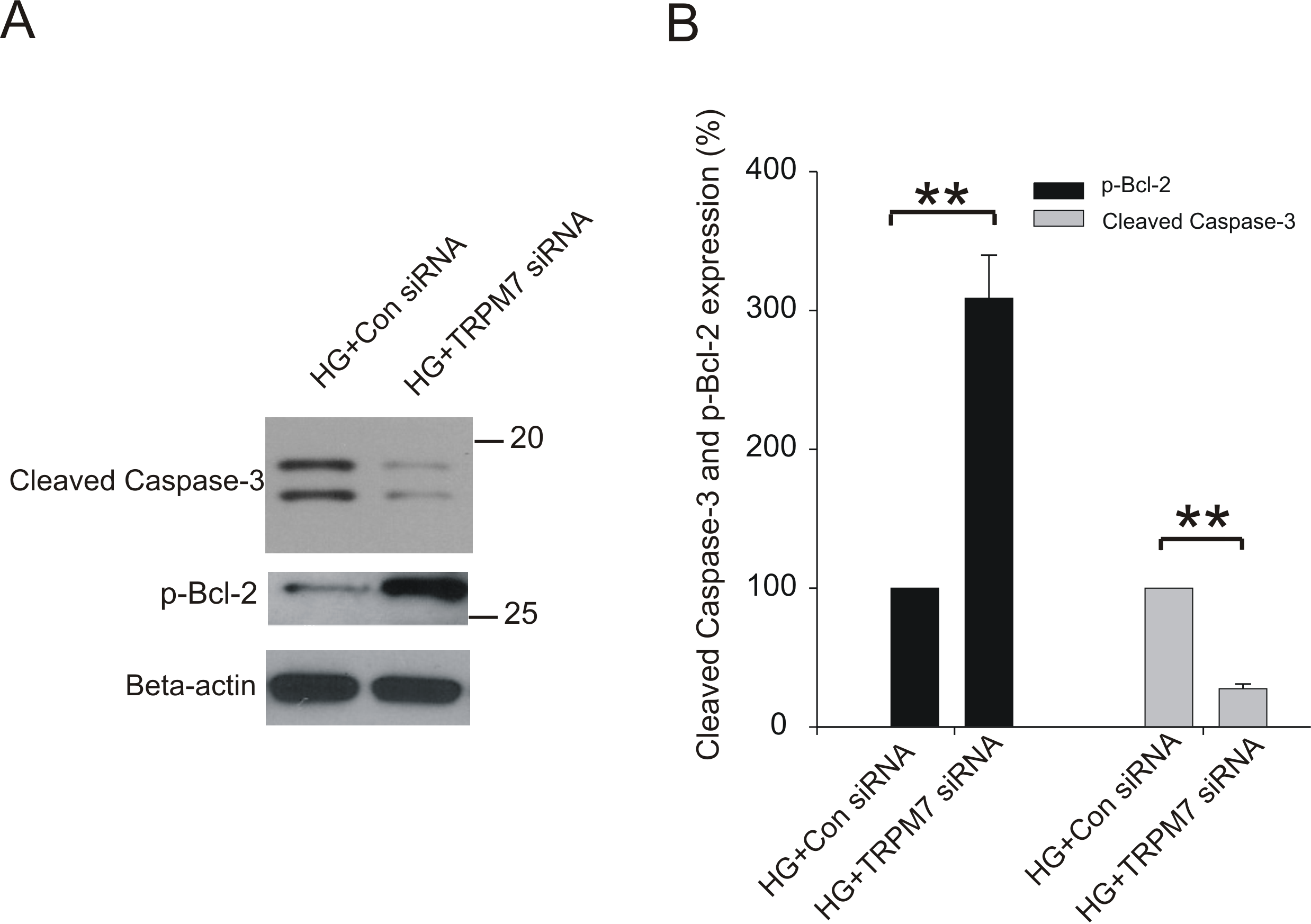

Supplement: Figure S3 — Effect of TRPM7 siRNA on the expression of cleaved Caspase-3 and phospho-Bcl-2 in HG treated HUVECs. The cells were preincubated with TRPM7 siRNA or control siRNA for 48h, and then stimulated with HG for 72h. Following that, Western blotting analysis was performed. (A) Representative blots and (B) Summary data showing the expression of cleaved Caspase-3 and phospho- Bcl-2. Data are mean ± S.E. (n=3, **p<0.01). (TIF) [file pone.0079540.s003.tif]
